# Population-based geographic access to endocrinologists in the United States, 2012

**DOI:** 10.1186/s12913-015-1185-5

**Published:** 2015-12-07

**Authors:** Hua Lu, James B. Holt, Yiling J. Cheng, Xingyou Zhang, Stephen Onufrak, Janet B. Croft

**Affiliations:** Division of Population Health, National Center for Chronic Disease Prevention and Health Promotion, Centers for Disease Control and Prevention, 4770 Buford Highway, N.E., Mailstop F-78, Atlanta, GA 30341 USA; Division of Diabetes Translation, National Center for Chronic Disease Prevention and Health Promotion, Centers for Disease Control and Prevention, Atlanta, GA USA; Division of Nutrition, Physical Activity and Obesity, National Center for Chronic Disease Prevention and Health Promotion, Centers for Disease Control and Prevention, Atlanta, GA USA

**Keywords:** Geographic access, Spatial coverage, Endocrinology, Geographic information system

## Abstract

**Background:**

Increases in population and life expectancy of Americans may result in shortages of endocrinologists by 2020. This study aims to assess variations in geographic accessibility to endocrinologists in the US, by age group at state and county levels, and by urban/rural status, and distance.

**Methods:**

We used the 2012 National Provider Identifier Registry to obtain office locations of all adult and pediatric endocrinologists in the US. The population with geographic access to an endocrinologist within a series of 6 distance radii, centered on endocrinologist practice locations, was estimated using the US Census 2010 block-level population. We assumed that persons living within the same circular buffer zone of an endocrinologist location have the same geographic accessibility to that endocrinologist. The geographic accessibility (the percentage of the population with geographic access to at least one endocrinologist) and the population-to-endocrinologist ratio for each geographic area were estimated.

**Results:**

By using 20 miles as the distance radius, geographic accessibility to at least one pediatric/adult endocrinologist for age groups 0–17, 18–64, and ≥65 years was 64.1 %, 85.4 %, and 82.1 %. The overall population-to-endocrinologist ratio within 20 miles was 39,492:1 for children, 29,887:1 for adults aged 18–64 years, and 6,194:1 for adults aged ≥65 years. These ratios varied considerably by state, county, urban/rural status, and distance.

**Conclusions:**

This study demonstrates that there are geographic variations of accessibility to endocrinologists in the US. The areas with poorer geographic accessibility warrant further study of the effect of these variations on disease prevention, detection, and management of endocrine diseases in the US population. Our findings of geographic access to endocrinologists also may provide valuable information for medical education and health resources allocation.

**Electronic supplementary material:**

The online version of this article (doi:10.1186/s12913-015-1185-5) contains supplementary material, which is available to authorized users.

## Background

The continued increase in US population, longer life expectancies, and the increase in the proportion of the population aged ≥65 years since 1950 [1], will result in Americans experiencing physician shortages of at least 91,500 in all medical specialties by 2020 [2]. An endocrinologist is a specialist in internal medicine or pediatric medicine who diagnoses and manages a wide range of diseases including diabetes, obesity, osteoporosis, and other disorders of the endocrine system. Diabetes affects 25.8 million people in the US [3] and one in three US adults are obese [4]. Clinical endocrinology is anticipated to be in greater demand by 2020 because of the larger proportion of the population with aging issues, obesity, and diabetes, and it has been suggested that this demand will exceed the capacity of the endocrinology workforce [5–8]. This prediction is supported by evidence of patients’ long wait times for the initial visit with an endocrinologist [6, 9]. The latest report from the 2012 Endocrinologist Survey showed that the average clinic waiting time was 37 days, and patients in some regions even experienced 3–6 month delays [10].

Our assessment of spatial accessibility incorporates separate notions of availability (e.g., the physical presence of endocrinologists who are available to treat patients) and accessibility (a consideration of impedance for patients to travel to the treatment locations) [11]. Accessibility, one of these dimensions for evaluating access to health care, describes geographical barriers including distance, transportation, travel time, and cost. It highlights the geographical location of services in relation to population in need [12, 13]. Geographic accessibility to endocrinologists is an important aspect of medical accessibility, which can affect the quality and timeliness of care for patients with metabolic and endocrine diseases [6]. While a study showed that the national ratio of children to pediatric endocrinologists in 2003–2004 was 290:1 for children with diabetes, and 17,741:1 for children with obesity [14], the geographic shortage in the adult endocrinologist workforce is unknown.

The main purpose of this study is to evaluate geographic coverage of, and access to, endocrinologists at different geographic levels by age group, urban/rural status, and distances for both youth and adult populations using the 2012 National Provider Identifier (NPI) Registry, which includes the service addresses of endocrinologists, and 2010 US Census block-level population data. To our knowledge, this is the first report to address geographic access to endocrinologist services at the national, state, and county levels in the United States.

## Methods

### Endocrinologist data

The NPI Registry is part of the Health Insurance Portability and Accountability Act of 1996 Administrative Simplifications standard which is issued by the Centers for Medicare and Medicaid Services through the National Plan and Provider Enumeration System [15]. Since December 2012, the NPI Registry data is released and updated weekly and provides timely information for individual physician and physician group practices. It has a unique 10-digit identification number for each covered health care provider regardless of whether he/she is in individual practice or in a group practice.

This study used NPI Registry data released on July 9, 2012 using health care taxonomy code Endocrinology, Diabetes & Metabolism 207RE0101X and Pediatric Endocrinology 2080P0205X [16]. After excluding physicians practicing in US territories and outside the US, the study included 6,501 adult endocrinologists with practice locations in the 50 states and the District of Columbia (DC). There were only 1,203 pediatric endocrinologists in 47 states and DC and no pediatric endocrinology practices were identified in Idaho, Montana and Wyoming in 2012. Each endocrinologist was geocoded to his/her practice location’s street address.

### Population counts

Population counts at the census block level were obtained from Census 2010 Summary File 1. The US census block is the smallest geographic census unit [17], which is typically bounded by visible features such as streets, roads, and streams, or by nonvisible boundaries such as selected property lines and city, township, school district, and county limits [18]. We used the census block as the initial geographic unit to calculate potential population geographic access to an endocrinologist and were able to aggregate data to higher geographic levels (census tract, county, state, national, and areas defined by urban/rural status). We retained 6,207,027 out of 11,155,486 census blocks in the analysis and excluded 4,948,459 uninhabited (44.4 %) census blocks in the 2010 US Census. Urbanized areas have been defined by the Census Bureau as areas consisting of multiple census blocks with combined populations equal to or greater than 50,000; urban clusters as areas with populations of at least 2,500 and less than 50,000; and rural areas as all other remaining areas [19]. Each census block is identified by the Census Bureau as belonging to an urbanized area, urban cluster, or rural area.

Census block internal geometric centroids were used to calculate distances to endocrinologist practice locations. The population counts of blocks were also stratified into three age groups: 0–17, 18–64 and ≥65 years. Those population counts were linked with their corresponding census block centroids. Because we did not have data on the proportion of pediatric patients who were treated by adult endocrinologists, we only used pediatric endocrinologists to estimate the accessibility to pediatric endocrinologists for children 0–17 years old, following the example of Lee (2008).

### Buffer zones around endocrinologist practice locations

**Fig. 1:**
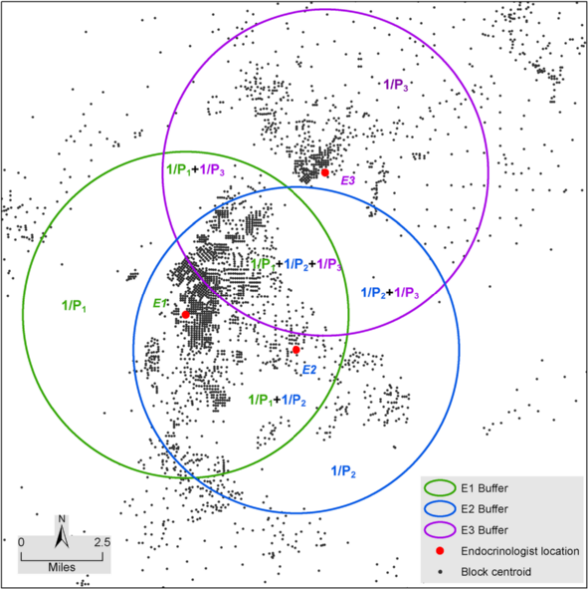
Illustration of calculation for each endocrinologist covered population and census block points covered by endocrinologists

Our general approach to estimating population geographic access to endocrinologists is broadly similar to that used in floating catchment area (FCA) spatial accessibility metrics [20–24]. The FCA metric most closely aligned to our approach is the two-step floating catchment area (2SFCA) [20]. 2SFCA provides a flexible approach to quantify population access to spatial opportunities within a predefined distance searching boundary. An important difference in our approach is that we used Euclidean distance between census block-level populations and endocrinologists to measure geographic accessibility. 2SFCA most commonly uses driving distances or driving times, but because our study was national and the basic unit of analysis was the census block level, this particular network analysis approach was computationally impractical. Instead, we chose to use the simpler Euclidean distance. By using each endocrinologist practice location as a center, we used a specific radius to create a circular area as a buffer zone (Fig. 1) and to identify the total population residing within that buffer zone. Extensions of the 2SFCA, such as the Enhanced 2SFCA [21], the Kernel Density 2SFCA [22], the Three Step FCA [23], and the Modified 2SFCA [24], specifically acknowledge variations in travel likelihood due to increased distances, and account for this by incorporating distance decay functions as weights. All these FCA methods adopted a single predefined distance search boundary, such as 15 miles or 30 or 60 travel/driving minutes. Considering variations in distance decay and population density across the US, particularly given the nature of urban/rural landscapes, we used a series of radii (5, 10, 15, 20, 30, and 50 miles) around each endocrinologist practice location to account for different transportation modes used to access endocrinologists. We assumed 5-mile and 10-mile distances to be reasonable proxies for travel by public transportation in urban areas; 15-mile and 20-mile distances to be approximate 20–30 min travel time by automobile; and a 50-mile distance to be approximately equal to 1 h of travel time by automobile, which may be more typical of travel time from a rural area to a regional medical center. If a census block’s centroid was inside a specific buffer zone of an endocrinologist’s location, all (100 %) of the population in that block was assumed to have access to that endocrinologist.

### Geographic access to an endocrinologist

We used three methods to calculate/illustrate geographic access to an endocrinologist. The first method was to estimate the coverage rate – the percentage of population within a geographic area (i.e., county, state, nation, or urban/rural area) that was within a specific radius distance or buffer zone for at least one endocrinologist. The second method was to estimate the total number of endocrinologists for a specified census block. And the third method was to calculate the population-to-endocrinologist ratio in a geographic area.

#### Estimating the coverage rate

For the first metric, we accounted for geographic access to endocrinologists, regardless of the political boundaries, with the only consideration being distance to endocrinologist practice locations. The circular buffer zone approach was used to estimate the number of the population with access to each endocrinologist within a defined geographic area [25] . We identified the population within and outside of a specific buffer zone to calculate the percentage of the population who had access to at least one endocrinologist for defined geographic areas (e.g., state, county, nation, urban/rural areas).

#### Estimating total number of endocrinologists for a specified census block

For the second metric, we equally divided an endocrinologist by the total number of persons (P_i_) in an endocrinologist-identified circular buffer zone; thus each person in that circular buffer zone has 1/P_i_ share of that particular endocrinologist. This step is conceptually the same as calculating the supply-to-demand ratio of the 2SFCA. Each person can be covered by multiple individual circular buffer zones of endocrinologists (i = 1, 2, 3, …, n), so the total number of endocrinologists covering a particular person at a block centroid location (E_b_) is the sum of 1/P_i_ of conjoined buffer zones, which is a measure of individual spatial accessibility to endocrinologists. This step is mathematically equivalent to the second step of the 2SFCA. Here, we aggregated for each census block all the buffers that contain that census block’s centroid. Since the census block is the smallest unit of census geography, the total number of shares of the endocrinologist for a census block will be the sum of the individual share multiplied by the total number of population P_b_ in that census block. For any geographic area (a) above the census block (e.g., county, state, or nation), the total number of endocrinologists is the sum of endocrinologists of all the blocks (b = 1, 2, 3… m) in that geographic area (Fig. 1).1).

$$ \begin{array}{l}{\mathrm{E}}_{\mathrm{b}}={\mathrm{P}}_{\mathrm{b}}\times {\sum}_{\mathrm{i}=1}^{\mathrm{n}}\left(\frac{1}{{\mathrm{P}}_{\mathrm{i}}}\right)\\ {}{\mathrm{E}}_{\mathrm{a}}={\sum}_{\mathrm{b}=1}^{\mathrm{m}}{\mathrm{E}}_{\mathrm{b}}\end{array} $$

a: specified geographic area (county, state, national, or urban/rural area)

b: specified census block

P_i_: total persons in a circular buffer zone around each endocrinologist

P_b_: total number of persons in a specified census block

E_b_: number of endocrinologists for a specified census block

E_a_: number of endocrinologists within a specified geographic area

n: number of endocrinologist buffer zones

m: number of blocks within a specified geographic area

#### Ratio of population to endocrinologist

Because our research is situated in public health practice, with a focus on the population, we calculated the population-to-endocrinologist ratio (R_a_). This ratio is the count of covered population per each endocrinologist for each geographic area within a specific circular buffer zone. More formally, it is the total covered population of a geographic area (P_a_) divided by the total number of the endocrinologists within that geographic area (i.e., R_a_ = P_a_/E_a_). In this study, we used this approach to examine the ratio by state, county, urban/rural areas, and distance or radius within specified buffer zones.

ArcGIS version 10.0 (Esri, Redlands, CA) with ArcGIS Online 10.0 North America Geocode Service was used to obtain the geographic coordinates (latitudes and longitudes) of endocrinologist practice locations, to calculate the access metrics, and to map the final results.

### Ethics statement

This study used only publically available data and did not constitute human subjects research.

## Results

In 2010, there were 74.2 million children aged 0–17 years, 194.3 million adults aged 18–64 years, and 40.3 million adults aged ≥65 years in the 50 states and DC (Table 1). The majority of the population lived in urbanized areas (71.7 % of children, 72.0 % of adults aged 18–64 years, and 66.8 % of adults aged ≥65 years). The majority of the US population had geographic access to at least one endocrinologist within 20 miles (64.1 %, 85.4 %, and 82.1 % for the respective age groups). Within 50 miles, the percentage of population with geographic access to at least one endocrinologist was 85.5 %, 96.6 % and 95.7 %, for the respective age groups. The ratio of population-per-endocrinologist within 20 miles for the population with geographic access was 39,492:1 for children, 29,887:1 for adults aged 18–64 years, and 6,194:1 for those aged ≥65 years at the national level. This varied by urban/rural area. For example, within 20-mile buffer zones, endocrinologist access was highest for children, adults aged 18–64, and adults aged ≥65 in urbanized areas (82.4 %, 98.6 % and 98.7 % respectively). The ratio was highest for children living in urbanized areas (40,234:1) while the ratio for adults was highest in urban clusters (92,983:1 for ages 18–64, 21,210:1 for ages ≥65) (Table 1).

**Table 1:** Selected characteristics of the study population, by age group: United States, 2012

Characteristic	0–17 years	18–64 years	≥65 years
	[*N* = 74,181,467]	[*N* = 194,296,087]	[*N* = 40,267,984]
Percentage of population urban/rural status			
Urbanized areas	71.7	72.0	66.8
Rural areas	18.7	18.8	22.5
Urban clusters	9.6	9.2	10.7
Cumulative percentage of age-specific population with access to at least one endocrinologist^a^ within a given distance (miles)			
5	24.8	58.6	55.5
10	44.5	74.2	70.0
15	56.3	81.0	77.0
20	64.1	85.4	82.1
30	73.8	91.3	89.3
50	85.5	96.6	95.7
Percentage of population with access to at least one endocrinologist^a^ within 20-mile buffer among all age-specific persons living in a specific urban/rural area			
Total	64.1	85.4	82.1
Urbanized areas	82.4	98.6	98.7
Rural areas	20.6	55.5	51.5
Urban clusters	11.3	43.9	42.7
Ratio of covered population to an endocrinologist^a^ within a 20-mile buffer			
Total	39,492:1	29,887:1	6,194:1
Urbanized areas	40,234:1	24,324:1	4,721:1
Rural areas	32,442:1	65,208:1	15,083:1
Urban clusters	32,018:1	92,983:1	21,210:1

^a^For children ages 0–17 years, estimates are calculated for access to at least one of 1,203 pediatric endocrinologists. For adults, the age-specific estimates are calculated for access to at least one of the same 6,501 adult endocrinologists

**Fig. 2:**
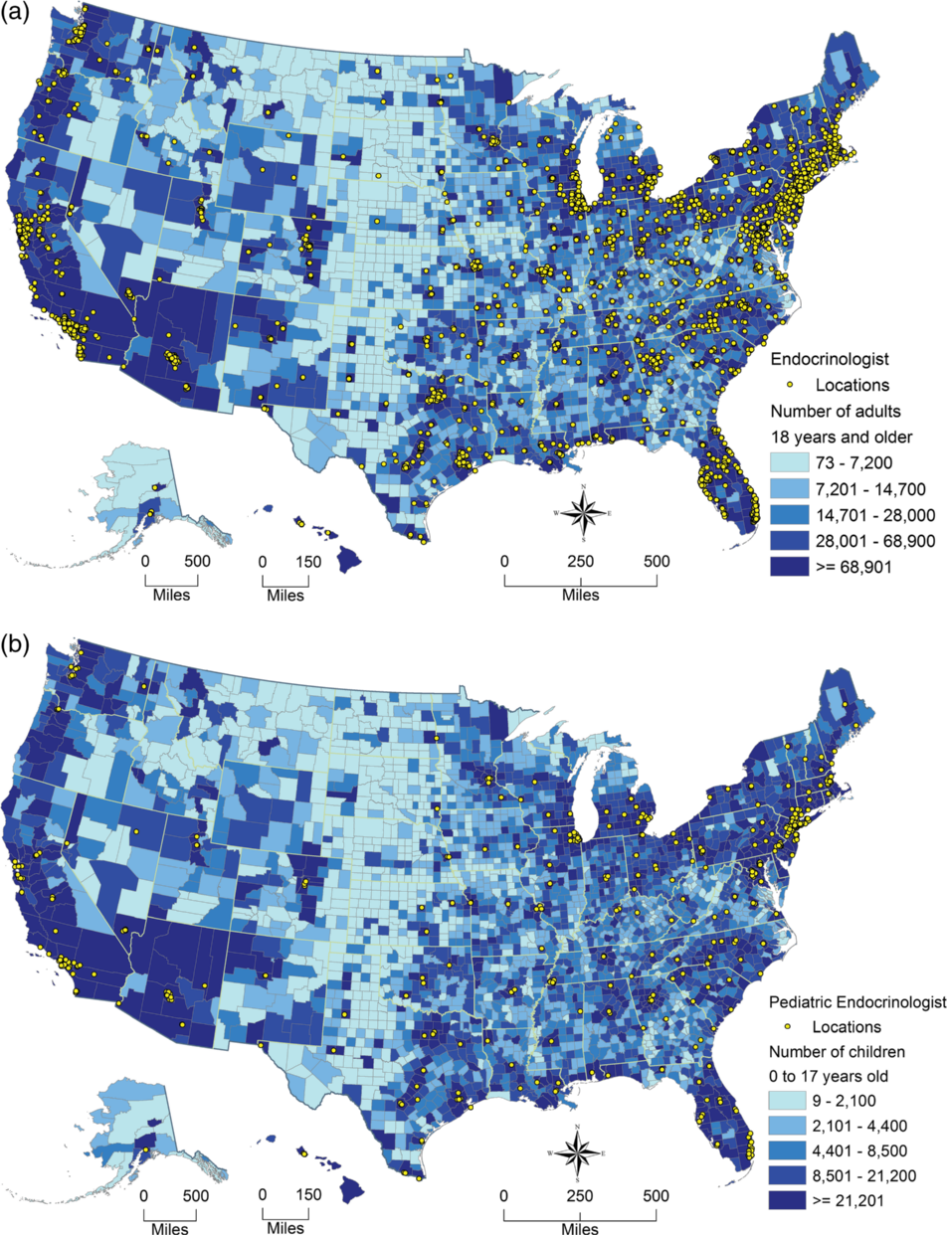
Endocrinologist locations overlaid with 2010 census population count by US counties. (**a**) Endocrinologists overlaid with adults aged ≥18 years and (**b**) Pediatric endocrinologists overlaid with children aged 0–17 years

Figure 2 shows two US maps of county-specific population counts for adults aged ≥18 years and for children. The locations of pediatric/adult endocrinologists are also depicted on the two age-specific maps. Endocrinologists were generally located in high-density populated areas, while urban clusters and rural areas had fewer endocrinologists. Among 6,501 adult endocrinologists, 6,201 (95.4 %) were located in urbanized areas, 176 (2.7 %) were located in urban clusters and 124 (1.9 %) were located in rural areas. Similarly among 1,203 pediatric endocrinologists, 1,165 (96.8 %) were located in urbanized areas, but only 17 (1.4 %) were located in urban clusters and 21 (1.8 %) were located in rural areas.

Within the 3,143 US counties or county-equivalents, 734 counties (whose population represented 78.7 % of the US population aged ≥ 18 years) had at least one adult endocrinologist practice and 233 (whose population represented 52.0 % of the US population aged 0–17 years) had at least one pediatric endocrinologist practice. Within 20 miles, in only 100 counties (whose population represented 17.7 % of the US population aged 0–17 years) did 100 % of the population aged 0–17 years have access to at least one pediatric endocrinologist, and in only 382 counties (whose population represented 42.4 % of the US population aged ≥18 years) did 100 % of the population aged ≥18 years have access to at least one endocrinologist. However, within a distance of 50 miles, 100 % geographic access was observed among 1,016 counties for children aged 0–17 years, 1,988 counties for adults aged 18–64 years, and 1,998 counties for adults aged ≥65 years. The number of counties without any access within 50 miles of an endocrinologist location was 1,146 (whose population represented 9.0 % of the US population aged 0–17 years) for children, 414 counties (whose population represented 1.5 % of the US population aged 18–64 years) for adults aged 18–64 years, and 419 counties (whose population represented 1.8 % of the US population aged ≥65 years) for adults aged ≥65 years.

**Fig. 3:**
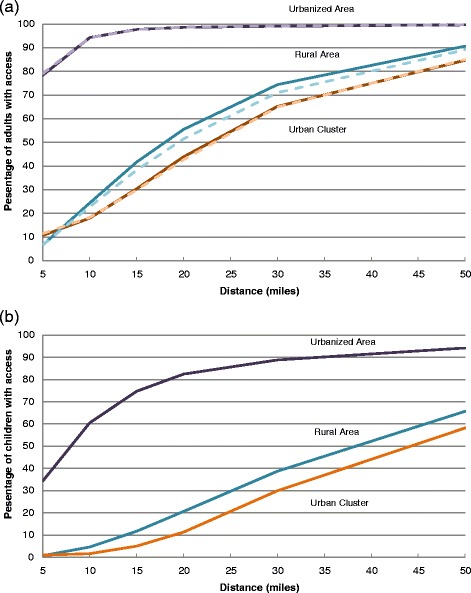
Percentage of US population with access to at least one endocrinologist. (**a**) Percentage of adults aged ≥18 years with access to at least one endocrinologist, by age group, urban/rural characteristics, and distance to endocrinologist locations. Age groups include 18–64 years (solid line) and ≥65 years (dashed line). (**b**) Percentage of children aged 0–17 years with access to at least one pediatric endocrinologist by urban/rural characteristics and distance to endocrinologist locations

Figure 3 shows the percentage of the age-specific populations that had access to at least one endocrinologist by urban/rural status and distance. The urbanized area adult populations (both aged 18–64 years and aged ≥65 years) had at least one endocrinologist at much shorter distances. Almost 99 % of adults living in urbanized areas had access to an adult endocrinologist within a 20-mile radius distance, while only about 53 % of the rural and 43 % of the urban cluster adults could access an adult endocrinologist within same distance. However, at a 50-mile distance, 90 % of rural adults and about 85 % of urban cluster adults had access to at least one endocrinologist. Contrasted with accessibility to adult endocrinologists, accessibility to pediatric endocrinologists was much more limited. Only about 80 % of urban, 20 % of rural, and 10 % of urban cluster children could access at least one pediatric endocrinologist within 20 miles. At a distance of 50 miles, over 94 % of urban, 66 % of rural, and 58 % of urban cluster children had access to at least one pediatric endocrinologist.

**Fig. 4:**
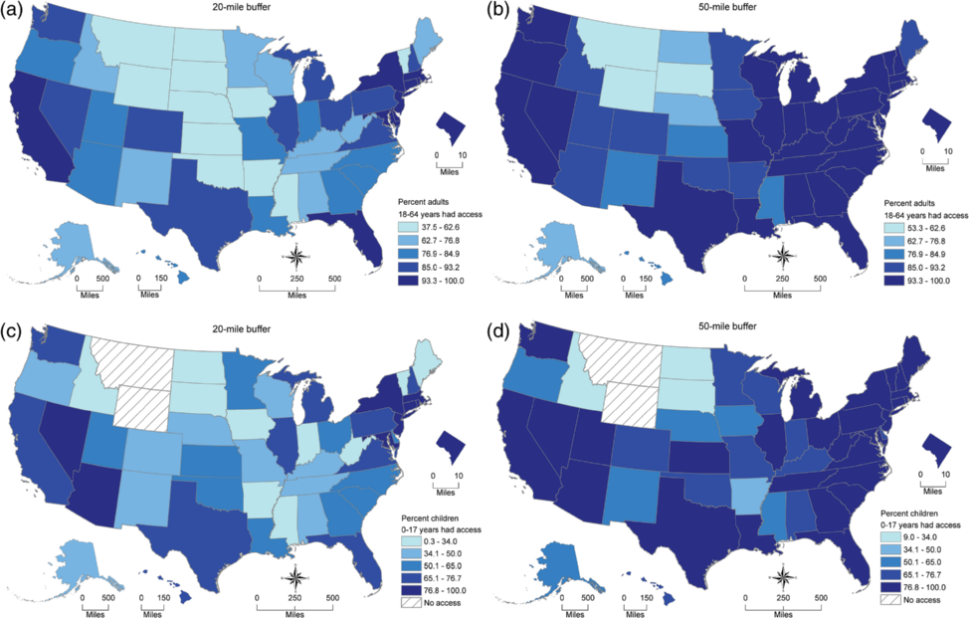
State-specific percentage of population with access to at least one endocrinologist by distance. (**a**) State-specific percentage of adults aged 18–64 years with access to at least one endocrinologist within 20 miles. (**b**) State-specific percentage of adults aged 18–64 years with access to at least one endocrinologist within 50 miles. (**c**) State-specific percentage of children aged 0–17 years with access to at least one pediatric endocrinologist with 20 miles. (**d**) State-specific percentage of children aged 0–17 years with access to at least one pediatric endocrinologist with 50 miles

Figure 4 shows four US maps of state-specific percentages of adults aged 18–64 years and children with access to an endocrinologist within 20 miles and 50 miles. Maps for adults aged ≥65 years are not shown because they are similar to those for adults 18–64 years. Adults aged 18–64 years had a coverage rate (within 20 miles) that ranged from 37.5 % in Wyoming to 100 % in 3 of the smaller states (Connecticut, New Jersey, Rhode Island) and DC. Expanding the distance threshold to 50 miles resulted in a range of 53.4 % in Wyoming to 100 % for 12 states (Connecticut, Delaware, Indiana, Kentucky, Maryland, Massachusetts, New Jersey, Ohio, Pennsylvania, Rhode Island, South Carolina, and Virginia) and DC. In 2012, there were no pediatric endocrinologists reported by the NPI Registry data in Idaho, Montana, and Wyoming. Idaho’s pediatric endocrinologist geographic access was due to the presence of these specialists in neighboring states. However, children in Montana and Wyoming had no access within a 50-mile distance from neighboring states. The state-specific percentages of children with access to at least one pediatric endocrinologist ranged from 0 % in Montana and Wyoming to 100 % in DC within 20 miles, and from 0 % in Montana and Wyoming to 100 % in Connecticut, New Jersey, Rhode Island and DC within 50 miles.

Detailed state-specific percentages of populations with access to at least one endocrinologist, by age group and distances in the US are provided in Additional file 1.

**Fig. 5:**
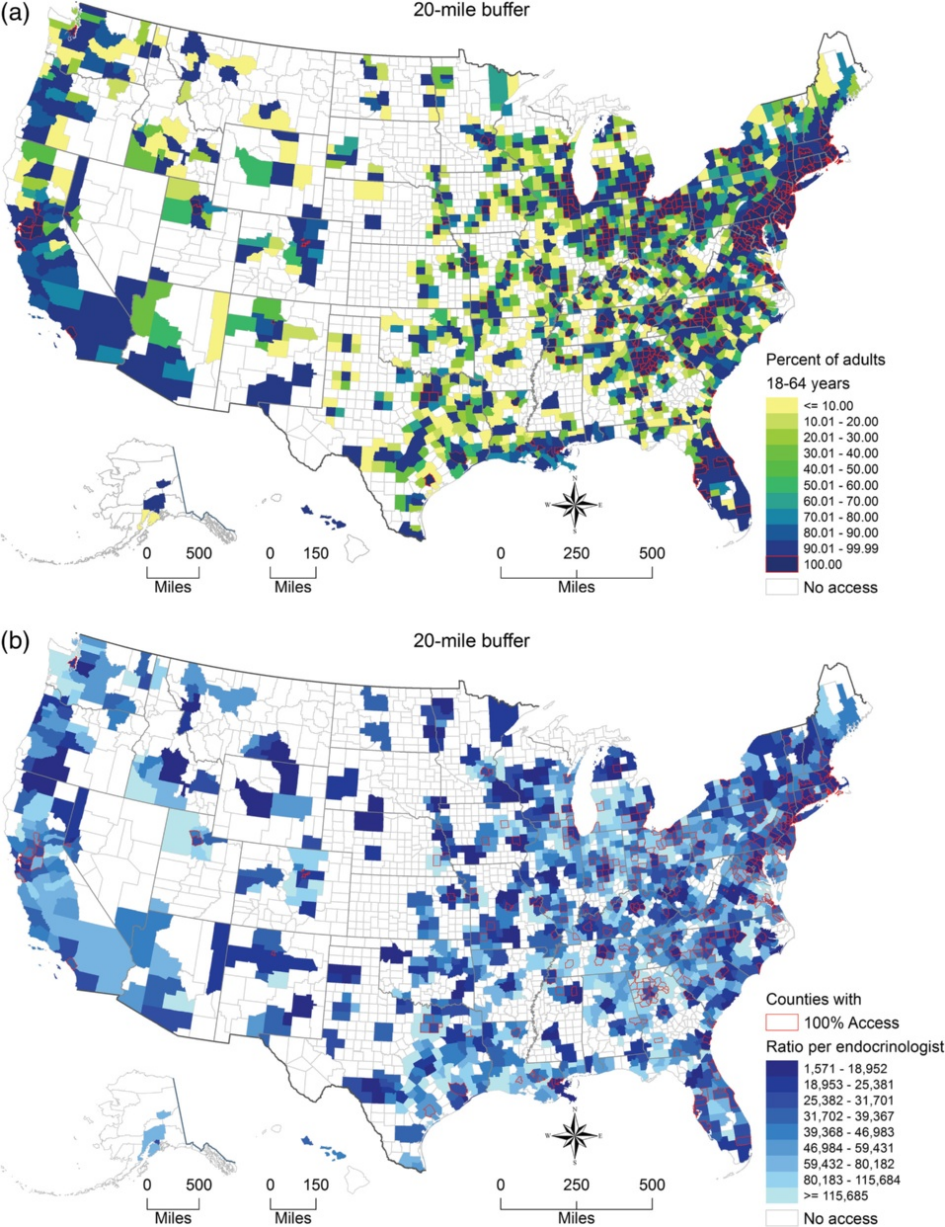
Endocrinologist accessibility for adults aged 18–64 years by US county, 2012. (**a**) Percentage of adults aged 18–64 years who had access to at least one endocrinologist with 20 miles. (**b**) Ratio of adults aged 18–64 years to endocrinologist for covered population within 20 miles

There were 1,966 (62.6 %) of 3,143 US counties whose population had some access to an adult endocrinologist within 20 miles for adults aged 18–64 years. Among them, 382 counties had 100 % geographic access. Figure 5a shows the county-specific percentage of adults aged 18–64 years with access to at least one adult endocrinologist within 20 miles. Figure 5b shows that the ratio of adults aged 18–64 years per endocrinologist ranged greatly between those 1,966 counties from 1,571:1 to 115,685:1 and greater.

**Fig. 6:**
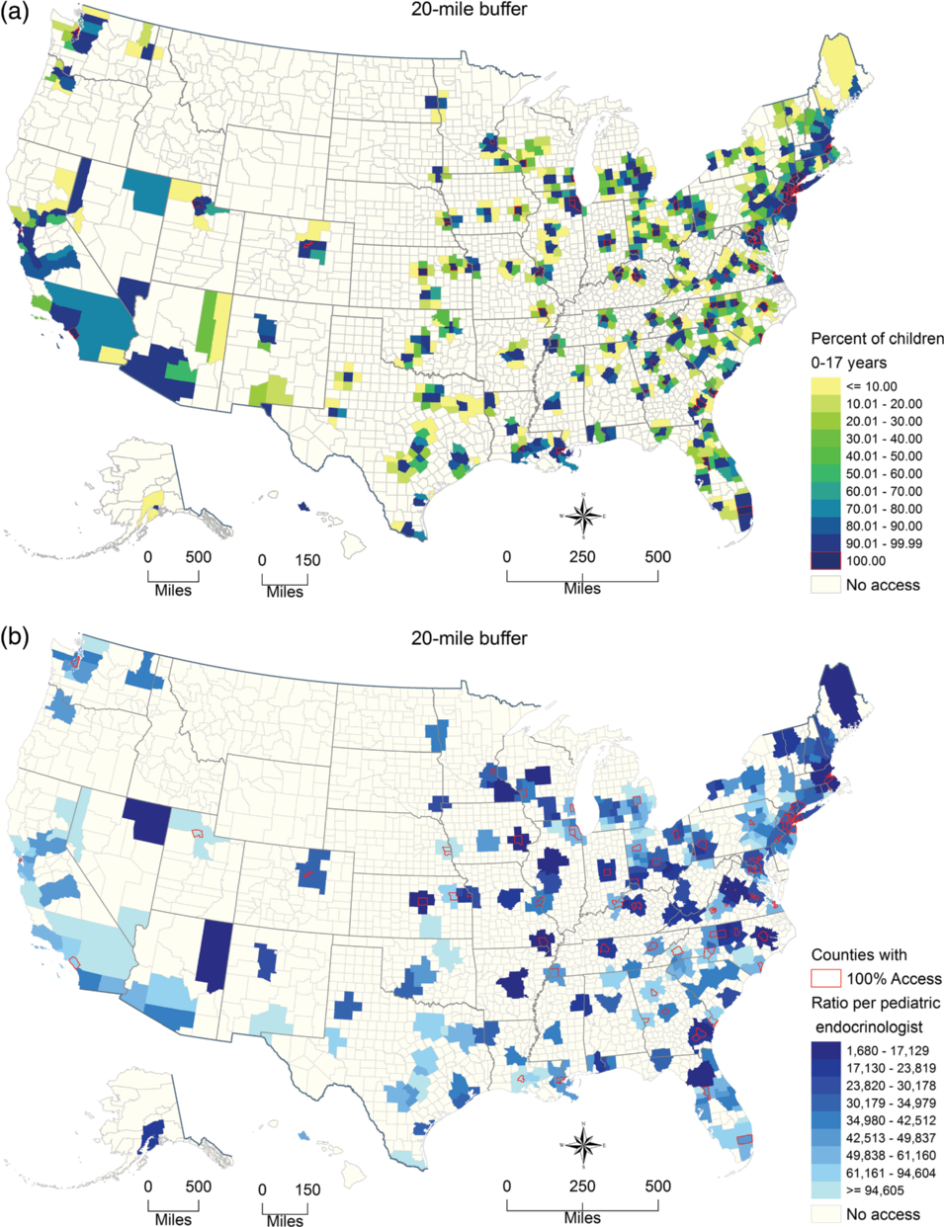
Pediatric endocrinologist accessibility for children aged 0–17 years by US county, 2012. (**a**) Percentage of children aged 0–17 years who had access to at least one pediatric endocrinologist within 20 miles (**b**) Ratio of children aged 0–17 years to pediatric endocrinologist for covered population within 20 miles

Figure 6a shows the county-specific percentages of children with access to at least one pediatric endocrinologist and Fig. 6b shows the county-specific ratios of children to pediatric endocrinologist within 20 miles.

Additional file 2 shows the county-specific percentage of adults aged ≥ 65 years with access to at least one adult endocrinologist within 20 miles.

## Discussion

By using the 2012 NPI Registry data and 2010 US census data linked with geographic locations, we provide a broad picture of the geographic access of the endocrinology workforce throughout the US at many different access distances. We found that 35.9 % of children, 14.6 % of adults aged 18–64 years, and 17.9 % of adults aged ≥65 years did not have access to any endocrinologist within a 20-mile radius. According to the US 2010 census, 71.2 % of the US population lives in an urbanized area, 9.5 % lives in urban clusters and 19.3 % lives in rural areas [19]. In urbanized areas, almost 99 % of US adults had access to at least one endocrinologist and 82 % of children had access to at least one pediatric endocrinologist within 20-miles of their census block centroid. However, adults and children living in rural areas and urban clusters required longer travel distances to gain access. Within 50 miles, about 90 % of adults in rural areas and 85 % in urban clusters had access to an endocrinologist but only about 65 % of children in rural areas and 60 % in urban clusters had access to a pediatric endocrinologist. These results were interesting in that populations living in urban clusters had lower spatial accessibility than populations living in rural areas. This is due to the nature of endocrinologist geographic distributions and the fact that urban clusters tend to be relatively far away from urbanized areas where most endocrinologists practice. Rural area populations that were near urbanized areas and urban clusters had spatial access to endocrinologists that practiced in them. Meanwhile, we found that urban cluster populations had less access to urbanized area endocrinologists. The population weighted share of endocrinologists increased more for rural areas than for urban clusters. These results also confirm earlier findings that there are considerable geographic disparities in the supply of pediatric endocrinologists [8, 14]. For each urban/rural area and distance, children had lower access to a pediatric endocrinologist in contrast to adult access to an adult endocrinologist. Because children are less likely to have endocrine diseases, the shortage may not be severe. Accessibility for both adults and children varied by state and county. Our findings on geographic access to endocrinologists may provide valuable information for medical education and health resources allocation.

The coverage approach using defined buffer zones in this study avoids the problem that arises when aggregating data according to existing census geographic units (e.g., states, counties), which vary greatly in size and do not necessarily contain homogeneous populations [26]. Our method also accounts for potential cross-boundary (county and state) health care seeking behaviors in the US. Traditional approaches for estimating geographic access of physicians generally are limited to those physician practices in a specific area, such as a county. That simple and intuitive traditional approach inappropriately assumes no cross-border health-providing or health-seeking behaviors outside of that geographic area. However, people very often seek health service by crossing political boundaries such as state or county lines especially when residing in smaller geographic areas, isolated rural areas, or in residences near state or county borders.

Our general approach is similar to the two-step floating catchment area (2SFCA) method of Luo and Wang [20]. In the 2SFCA method, most commonly a single driving time distance between physician practice locations and populations is used to define the catchment area (buffer zone). We explored the multiple buffer distance zones to explore the sensitivity of spatial access coverage to multiple distance search boundaries. Also, we used Euclidian distance to create a buffer zone (catchment area) because of the significant computation time for a national level study, and because for a non-emergency service, both straight-line distance and drive time/distance provide similar precision [27]. By using our coverage approach, we virtually divided an endocrinologist by the population within that zone as a share (i.e., a physician-to-population ratio for the physician) and distributed a share to each person living in that circular buffer zone. The total accessibility for an individual to any physicians is the total shares connected to that individual. In this way, the accessibility of a geographic area is estimated by the total population in that area divided by the sum of the total shares of all individuals in the interested area. As with other FCA methods, our approach allows flexibility in computing estimates for different geographic levels and for calculating large nation-wide estimates. We did not overcome the subjectivity of a predefined distance search boundary like all the family of 2SFCA methods. The distance decay function is applied to some modified 2SFCA methods to account for the unequal accessibility to physicians within the single predefined distance searching boundary or travelable distance limit. Like classic 2SFCA, we did not consider the unequal probability of access to endocrinologists within a predefined distance buffer zone, because to our knowledge, for non-emergency care access to physicians, distance decay was limited within a travelable distance or predefined distance buffer zone in this study. Since we do not have an informed knowledge of travelable distances that would work for our entire study area, we chose to implement the conceptually simpler approach of using multiple distance buffers as a sensitivity analysis.

Endocrinologists are a crucial part of the health care network required to meet the growing need for the diagnosis and management of complex cases of obesity-related diseases such as diabetes and metabolic syndrome. Unfortunately, the prevalence of obesity in the US has increased three-fold among children and nearly as greatly among adults during the past three decades to become a critical public health problem [4, 28]. As of 2011–2012, the prevalence of obesity remained high among children aged 2–5 years (8.4 %), aged 6–11 years (17.7 %), and aged 12–19 years (20.5 %) [29]. Likewise, more than one-third (35.4 %) of US adults aged ≥20 years were obese during the same time period [4]. Concurrent with the rise in obesity, the prevalence of diagnosed diabetes among adults has also increased from 3.5 % in 1980 to 9.0 % in 2011 [30]. Among children, a 2009 study estimated that there were 187,000 children with diabetes in the US who were potentially in need of consultation with a pediatric endocrinologist [31]. Future studies would benefit in estimating geographic access of obese and diabetic adult populations to endocrinologists and in updating the seminal work by Lee et al., which used data from the 2003–2004 National Survey of Children’s Health to estimate the ratios of diabetic children and obese children to the 2004 supply of pediatric endocrinologists in the US [14].

There are some limitations in this study. First, the NPI Registry data from the Centers for Medicare and Medicaid Services may include more endocrinology practices than other physician workforce datasets because of the HIPAA requirement that all covered health care providers must obtain an NPI [15]. Other reports on issues related to the endocrinologist workforce have used the American Medical Association Masterfile [5, 9, 10], American Board of Pediatrics records [14], American Board of Internal Medicine records [6, 7], and membership records of The Endocrine Society [32]. Therefore, our estimates may overestimate actual numbers of endocrinologists in practice. Second, we have not accounted for board certification status, excluded retired or inactive professionals, or identified the major professional activity (i.e., proportion of time spent in office-based practice, hospital staff, research, teaching, or administrative duties, multiple practice) as other reports have done [5, 14]. Vigersky and colleagues [10] estimated that there were 5,496 board certified adult and 1,016 pediatric endocrinologists in 2011. Furthermore, because NPI only included one practice location for physicians, we were unable to include endocrinologists’ multiple practice locations in our analysis. Therefore, we may have either under- or over-estimated the geographic accessibility to endocrinologists in this study. Third, since we used Euclidean distance as a proxy for travel distance, our metric may overestimate the geographic accessibility to an endocrinologist, especially in rural or mountain areas where road networks are more limited than in urban areas. Actual travel distances or travel times using network analysis may be able to improve precision. However, for a national study, it would be computationally prohibitive at present to analyze coverage using detailed street network data. In addition, a recent analysis showed that for nonemergency travel to hospitals, the added precision of using street networks is inconsequential [27].

A further limitation is that it is possible that there are important variations in practice patterns of endocrinologists and primary care physicians who treat patients with diabetes and obesity. Such practice variations could mitigate lack of access to endocrinologists, particularly in areas or cases where less severe presentations are treated by primary care physicians and/or other health specialists, such as dietitians. Information on practice variations could be useful in further research on the association between geographic variations in access to care and health outcomes. In addition, like classic 2SFCA, we did not consider the unequal probability access to endocrinologists within a predefined distance buffer zone. We assumed that for the non-emergent care access, distance decay was limited within a predefined buffer zone.

A clear picture of the current geographic access to endocrinologists in the US is important for resource planning and allocation and intervention strategy development. Residents of urban clusters and rural areas are likely to continue to travel greater distances to reach an endocrinologist. According to Vigersky [10] 85 % of diabetes care was performed by health care providers other than endocrinologists (e.g., primary care physicians, physician assistants, nurse practitioners). One solution that has been recommended to fill the gaps in geographic access, particularly for children with diabetes, is to promote the development and/or improvement of telemedicine opportunities so that primary care providers can manage the patient but consult with specialists [9].

## Conclusions

There were substantial nationwide variations in geographic accessibility to pediatric and adult endocrinologists at county and state levels, as well as disparities in access to an endocrinologist within a reasonable driving distance, by urban/rural status and by age. Our findings show an unequal geographic distribution, including shortages, of the endocrine workforce in the US, and highlight areas with limited endocrinologist resources. Given the backdrop of increases in obesity and diabetes, understanding that geographic access to one of the types of health care providers (endocrinologists) who treat these conditions can inform future research by suggesting inclusion of geographic access to care as a potential explanatory variable for variations in obesity and diabetes. Our approach can be used to further assess the impact of geographic variations in access to endocrinologists on disease detection, prevention, and control. These methods also can be applied to other health care workforce spatial accessibility and coverage analyses.

## Additional files

Additional file 1:
**State-specific percentages of populations with access to at least one endocrinologist, by age group and distances in the United States.** (XLSX 20 kb) Additional file 2:
**Endocrinologist accessibility for adults aged ≥65 years by US county, 2012.** (a) Percentage of adults aged **≥**65 years had access to at least one endocrinologist with 20 miles. (b) Ratio of adults aged **≥**65 years to endocrinologist for covered population within 20 miles. (PNG 1302 kb)
