# Supplementary figures and images for: Population-based geographic access to endocrinologists in the United States, 2012
Source: BMC Health Serv Res. 2015 Dec 7;15:541. doi: 10.1186/s12913-015-1185-5 (PMC4672571; doi:10.1186/s12913-015-1185-5)

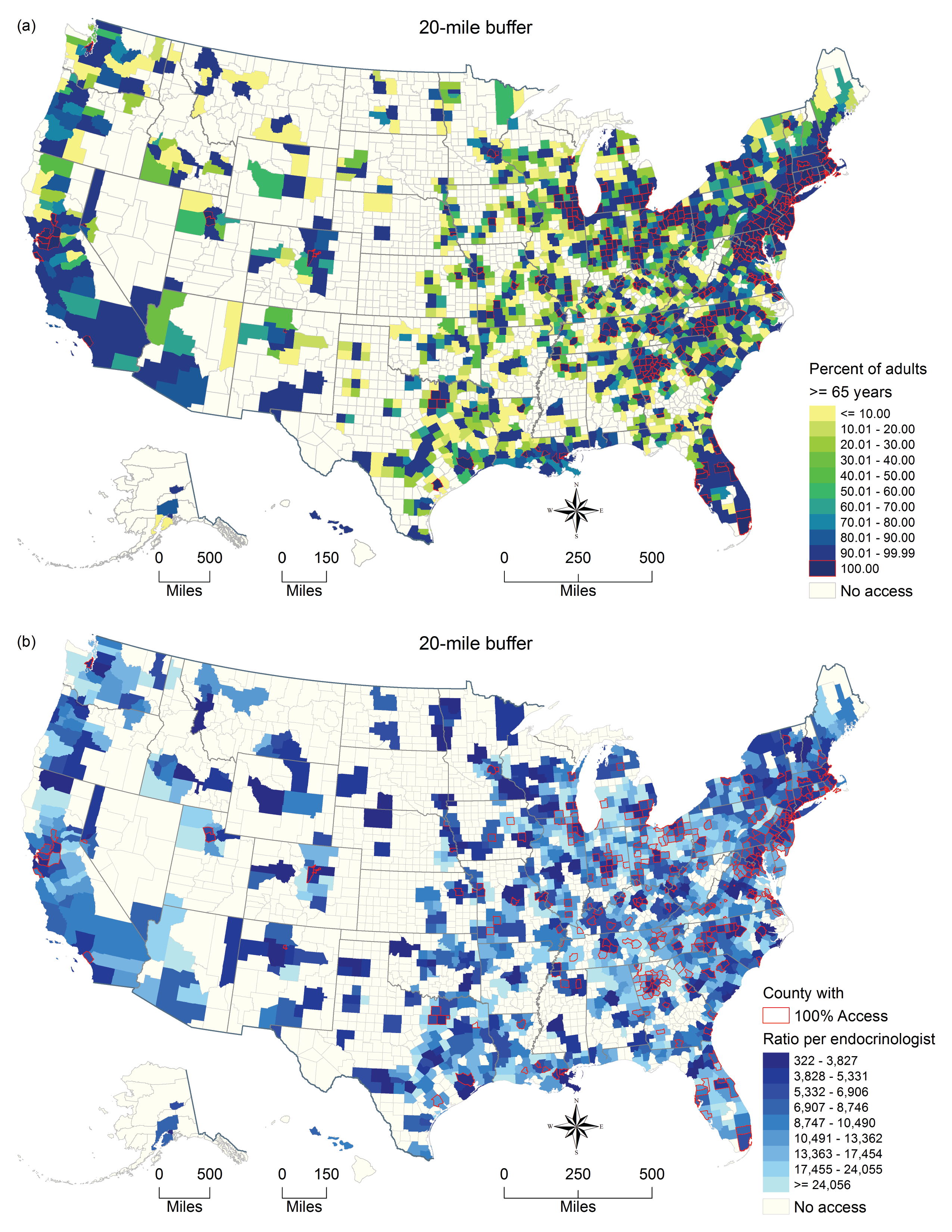

Supplement: Additional file 2: — Endocrinologist accessibility for adults aged ≥65 years by US county, 2012. (a) Percentage of adults aged ≥65 years had access to at least one endocrinologist with 20 miles. (b) Ratio of adults aged ≥65 years to endocrinologist for covered population within 20 miles. (PNG 1302 kb) [file 12913_2015_1185_MOESM2_ESM.png]
